# Effect of Muscular Exercise on Patients With Muscular Dystrophy: A Systematic Review and Meta-Analysis of the Literature

**DOI:** 10.3389/fneur.2020.00958

**Published:** 2020-11-12

**Authors:** Silvia Gianola, Greta Castellini, Valentina Pecoraro, Marco Monticone, Giuseppe Banfi, Lorenzo Moja

**Affiliations:** ^1^Unit of Clinical Epidemiology, IRCCS Istituto Ortopedico Galeazzi, Milan, Italy; ^2^Department of Laboratory Medicine and Pathological Anatomy, Ospedale Civile S. Agostino Estense, Modena, Italy; ^3^Department of Medical Sciences and Public Health, University of Cagliari, Cagliari, Italy; ^4^Neurorehabilitation Unit, Department of Neuroscience and Rehabilitation, G. Brotzu Hospital, Cagliari, Italy; ^5^IRCCS Istituto Ortopedico Galeazzi, Scientific Director, Milan, Italy; ^6^Università Vita e Salute San Raffaele, Milan, Italy; ^7^Department of Biomedical Sciences for Health, University of Milan, Milan, Italy

**Keywords:** exercise, muscular dystrophy, randomized controlled trial, systematic review, meta-analysis, physical therapy, rehabilitation, clinical decision-making

## Abstract

**Background:** Muscular dystrophy causes weakness and muscle loss. The effect of muscular exercise in these patients remains controversial.

**Objective:** To assess the effects of muscular exercise vs. no exercise in patients with muscular dystrophy.

**Methods:** We performed a comprehensive systematic literature search in the Medline, Embase, Web of Science, Scopus, and Pedro electronic databases, as well as in the reference literature. We included randomized clinical trials (RCTs) that reported the effect of muscular exercise on muscle strength, endurance during walking, motor abilities, and fatigue. Data were extracted independently by two reviewers. Mean difference (MD) and 95% confidence intervals (CI) were used to quantify the effect associated with each outcome. We performed pairwise meta-analyses and trial sequential analyses (TSA) and used GRADE to rate the overall certainty of evidence.

**Results:** We identified 13 RCTs involving 617 patients. The median duration of exercise interventions was 16 weeks [interquartile range [IQR] 12–24]. In the patients with facio-scapulo-humeral dystrophy and myotonic dystrophy, no significant difference in extensor muscle strength was noted between the exercise and the control groups [four studies, 115 patients, MD 4.34, 95% CI −4.20 to 12.88, *I*^2^ = 69%; *p* = 0.32; minimal important difference [MID] 5.39 m]. Exercise was associated with improved endurance during walking [five studies, 380 patients, MD 17.36 m, 95% CI 10.91–23.81, *I*^2^ = 0; *p* < 0.00001; MID 34 m]. TSA excluded random error as a cause of the findings for endurance during walking. Differences in fatigue and motor abilities were small. Not enough information was found for other types of dystrophy.

**Conclusions:** Muscular exercise did not improve muscle strength and was associated with modest improvements in endurance during walking in patients with facio-scapulo-humeral and myotonic dystrophy. Future trials should explore which type of muscle exercise could lead to better improvements in muscle strength.

**PROSPERO:** CRD42019127456.

## Introduction

Muscular dystrophies are a heterogeneous group of progressive, inherited diseases caused by mutations in genes involved in muscle function (1). Though they can vary widely in etiology and presentation, nearly all forms of muscular dystrophy cause muscle weakness and muscle loss, which may result in limitations of daily activities, and fatigability (2, 3).

Currently, there is no cure for muscular dystrophies. Treatment consists of medication, surgery and/or rehabilitation, including physical and muscle training, aerobic capacity training or aids and adaptations such as arm supports to enable performance of daily activities (4).

Whether patients with muscular dystrophies can benefit from muscular exercise remains debated. Physical exercise can have numerous psychological and physiological positive effects for the general population, such as improvements in self-estimate and plasma endorphin concentrations (5, 6). But because of muscle degeneration in muscular dystrophy, there may be the risk of exercise-induced adverse effects such as overwork weakness following supramaximal, high-intensity exercise (7). Guidelines for the prescription of physical exercise are based on low-quality evidence, which limits their confidence in strengthening and aerobic fitness training programs (7).

Randomized clinical trials (RCT) assessing the efficacy of muscular exercise for muscular dystrophies have been limited and inconclusive to date. These diseases are rare, and research possibilities are limited (8); nevertheless, recent trials have added data to the evidence base. We conducted an updated systematic review and meta-analysis of existing RCTs to further explore the effect of muscular exercise in patients with muscular dystrophy.

## Methods

### Registered Protocol and Reporting Guidelines

The systematic review protocol was registered with the International Prospective Register of Systematic Reviews database (PROSPERO identifier: CRD42019127456). We followed the Preferred Reporting Items for Systematic Reviews and Meta-Analyses (PRISMA) Statement guidelines for reporting (9) (Supplementary Material).

### Eligibility Criteria

For this systematic review we included only RCTs (both parallel-group and cross-over design RCTs) that enrolled patients with Duchenne's muscular dystrophy (DMD), Becker's muscular dystrophy (BMD), limb-girdle dystrophy (LD), facio-scapulo-humeral dystrophy (FSHD), and myotonic dystrophy (MyD). For the exercise intervention, we were interested in any muscular exercise as the core intervention, assessed on the basis of muscle strengthening or physical capacity and expressed as peak torque of strength, endurance during walking, motor abilities, and fatigue. All kinds of strength training, including aerobic cycling, fitness, weights, weight machines or elastic cords, were eligible for inclusion (10).

As control, trials were eligible irrespective of the type of control, with the caveat that any type of exercise or other type of intervention that would have limited our ability to separate and understand the role of muscular exercise was excluded. Control categories encompassed no intervention at all and usual care. We excluded studies in which the non-exercised limb was a control so as to avoid the cross-education phenomenon, since exercise of one side of the body can increase the voluntary strength of the contralateral side (11). Eligibility was not restricted by language, type of publication or patient age.

### Search Strategy

Two methodologists conducted the search strategy in the electronic databases: Medline (since 1966), Embase (since 1974), Web of Science (since 1950), Scopus (since 1996), and Pedro (since 1999). The last search was run in February 25 2019 and it was up to date in February 4, 2020. Reference lists of relevant studies were screened for further publications. In addition, www.ClinicalTrials.gov was investigated for ongoing trials.

### Outcomes

The primary outcomes were changes in muscle strength and endurance during walking. Muscle strength, measured with a dynamometer, is considered a measure of efficacy that captures changes in specific muscle groups and reflects optimal test conditions (12). Where authors reported outcome data for more than one muscle group, we extracted data according to a priority list: knee extensors, knee flexors, elbow flexors, elbow extensors, wrist flexors, and wrist extensors. Because this might be seen as a narrow outcome with limited everyday life value, we included endurance during walking as an outcome since it better represents the potential effectiveness of exercise training in ambulant dystrophic patients and mimics daily real-world conditions (13). Endurance during walking, as measured by tests such as the Six Minute Walking Test (6 MWT), was defined as the ability of a muscle to maintain its function or performance capacity over time and multiple contractions (14).

Secondary outcomes were motor abilities and fatigue. Motor ability was defined as the successful performance of motor skill based on a unit measure such as time or score (e.g., standing from supine) (15). Fatigue was defined as the inability of a muscle to generate force or power. It is an important limiting factor of exercise performance and muscle functional capacity (e.g., BORG scale) (16).

The end of treatment and follow-up assessment were used for each trial in the meta-analysis. All adverse events reported by the studies were recorded. When we identified relevant missing or unpublished data, we contacted the corresponding author of the primary study and requested the information.

### Data Collection and Extraction

Two researchers independently screened the studies for eligibility by title and abstract. Full texts were then evaluated for inclusion. Two authors independently extracted and entered the data from the studies onto data extraction forms. The information was reported in a table of main features: (i) characteristics of trial participants (age, type of dystrophy, disease stage, and muscle involved); (ii) characteristics of studies (study design, study year, country, sample size calculation, funding); (iii) outcomes (muscular strength, endurance during walking, motor abilities, and fatigue). A description of key elements of interventions, taken from the Template for Intervention Description and Replication (TIDieR) checklist, was recorded (17). Disagreement between reviewers was resolved by consensus; a third author was consulted if no agreement could be reached.

### Risk of Bias Assessment

The Cochrane Collaboration's tool was used to assess the risk of bias in the RCTs for random sequence generation, allocation concealment, blinding of participants and health care personnel, blinded outcome assessment, incomplete outcome data, and selective reporting.

### Overall Certainty of Evidence

Two authors independently assessed the certainty of evidence for the primary outcomes using the Grading of Recommendation, Assessment, Development and Evaluation (GRADE) framework methodology (18). Five GRADE domains—study limitations, consistency of effect, imprecision, indirectness, and publication bias—were analytically assessed. For imprecision, our assessment was informed by the findings of trial sequential analysis (TSA) (19). The final judgement on certainty was revised and downgraded by one or two levels as appropriate, reflecting the extent of bias in important quality domains. All reasons for rating down are reported in detail in Table 2. We used GRADEpro software (Tool) to present the study findings^1^.

### Statistical Analysis

To quantify the effect associated with each outcome, we used the mean difference (MD) or standardized mean difference (SMD) with 95% confidence intervals (CIs). We used the mean change from baseline scores since muscular dystrophies are rare diseases and we expected to encounter studies with small sample size and small effects. Imbalances between groups at baseline are therefore possible, even when randomization is adequately implemented (20). Positive effect measures indicated that muscle exercise is favored over no exercise for strength, endurance during walking, and motor abilities, while for fatigue, a negative effect measure indicated that the treatment was associated with less fatigue.

Meta-analyses were developed according to type of dystrophy (e.g., DMD and BMD split from the others). Heterogeneity was evaluated using the *I*^2^ statistic (21). To investigate potentially different effects on strength and endurance during walking, the studies were sub-grouped by group of muscles exercised (e.g., knee extensors). However, the overall interpretation of results, GRADE assessment of certainty, and TSA were derived from the overarching meta-analysis of all studies that assessed the outcome of interest.

We conducted a sensitivity analysis of the primary outcomes based on risk of bias for blinding of outcome assessor (detection bias: high risk vs. low and intermediate risk studies) and only published data. All analyses were performed using Review Manager (RevMan5) software version 5.3^2^.

We performed TSA to limit the risk of potential spurious conclusions from underpowered meta-analysis and repetitive significance testing. In detail, we can control the risk of I type and II type errors and introduce the calculation of a required information size by applying trial sequential monitoring boundaries, which can inform about the research needed to achieve conclusive evidence (22). TSA for continuous outcomes is possible, however, only when studies that use the same outcome measure and effect sizes are cumulated using MD, since expected standardized mean differences are prone to providing unrealistic information size (23). For muscle strength, we arbitrarily selected the peak torque measurement of knee extensor muscles to generate inference on the conclusiveness of findings. We estimated the diversity adjusted required information size (DARIS) based on the standard deviation observed in the control group of trials with low risk of bias (alpha 5%, beta 20%), and the observed diversity in the trials in the meta-analysis. We selected the highest quality trial (24), assuming a minimal important difference (MID) of 5.39 N, estimated by multiplying the effect size of 0.5 by the pooled standard deviation between groups (25). For endurance during walking, in order to avoid clinical heterogeneity, we performed a meta-analysis and a TSA only for the 6 MWT, the most common, reliable and feasible test in clinical trials (26). The 6 MWT measures the distance in meters walked in 6 min, wherein a greater distance indicates better performance. We estimated the DARIS by selecting a commonly reported MID of 34.3 m (27); we assumed a diversity of 50% among the meta-analyzed trials. We used TSA software beta version 0.9.5.5 (23).

## Results

The literature search identified 5,528 references, excluding duplicates. After screening and selection, 10 parallel group trials were included in the analysis (24, 28–36) and 1 cross-over trial (37). Two additional ongoing trials were also identified that were described only qualitatively. The study selection flow is illustrated in eFigure S1. The list of excluded published and ongoing studies are reported in eTables S1a,b.

Overall, 584 participants were involved (range 13–255). The majority (60.4%) had MyD, followed by FSHD (30.3%), and DMD (9.2%). All trials were conducted in Western countries between 1995 and 2018. A description of the interventions is presented in eTable S2. The duration of exercise intervention ranged from 8 to 52 weeks (median 16 weeks). Table 1 presents the characteristics of the trials. Most were judged as having a low risk of bias. Details are presented in eFigure S2.

**Table 1:** Study characteristics.

**References**	**Country**	**Mean Age ± SD**	**No. randomized**	**Muscular dystrophy type**	**Intervention and control groups**	**Trial duration (weeks)**	**Outcome**			
							**Muscle strength**	**Endurance**	**Motor abilities**	**Fatigue**
Lindeman et al. (28)	Netherlands	E:40 ± 11 C:37 ± 10	28	MyD	E: Exercise C: Usual care (no intervention)	24	Isokinetic peak torque, knee extensor and flexor muscles (Nm)	Endurance test 80% (s)	Standing up (s); descending and climbing stairs (sec); walking fast and comfortably (s)	-
Van der Kooi (29, 38)	Netherlands	E:36 ± 9 C:39 ± 9	65	FSHD	E: Exercise C: Usual care (no intervention)	52	MVIC, elbow and ankle muscles (N)	Isometric endurance	Timed motor performance tasks	Fatigue CIS checklist
Kierkegaard et al. (30)	Sweden	44 ± 11	35	MyD type1	E: Exercise C: Usual care (no intervention)	14		6 MWT (m)	1. TST (s) 2. TUG test (s)	BORG RPE score 0–20
Aldehag et al. (37)	Sweden	44 ± 11	35	MyD type1	E: Hand training C: No intervention	12	Isometric grip force, wrist and/or hand muscles (N)	1. Hand grip force (N) 2. Pinch grip force (N)	AMPS for (I-ADL)	-
Alemdaroglu et al. (33)	Turkey	9.5 ± 1.38	24	Early-stage DMD	E: Arm ergometer training C: Exercise at home	8	Isometric force, upper extremity muscles (N)	1. Unilateral placing (s) 2. Bilateral turning (sec) 3. Grip Strength (kg/f)	1. Standing from supine (s) 2. T-shirt donning (s) 3. T -shirt removing (s)	-
Andersen et al. (34)	Denmark	E: 45.7 (22-63)^*^ C: 51,3 (24-65)^*^	23	FSHD type 1	E: Aerobic training by bike C: No intervention	12	Isometric force, knee extensor, elbow extensor and flexor muscles (N)		1. FTSTS 2. 14-step-stair-test 3. Standing balance test	Score 0–10
Andersen et al. (35)	Denmark	E:53 ± 15 C:46 ± 9	13	FSHD type 1	E: High intensity training C: Usual care	8	Isometric force Hip flexor, knee extensor and knee flexor, and elbow flexor muscles (N)	6 MWT (m)	FTSTS	Score 0–10
Bankole et al. (24)	France	E:41 ± 9 C:40 ± 13	19	FSHD	E: Strength, and aerobic training C: Control	24	Isokinetic peak torque, knee extensor muscles (Nm)	1.6 MWT (m) 2. Number of repetition quadriceps	-	Fatigue severity scale
Jansen et al. (31)	Netherlands	10.5 ± 2.6	30	DMD	E: Assisted bicycle training of the legs and arms C: No intervention	24	MRC scale multiple muscle groups	Assisted 6-Min Cycling Test, leg and arm (s)	MFM	-
Voet et al. (32)	Netherlands	E:59 (21-68)^**^ C:52 (20-79)^**^	57	FSHD type 1	E: AET C: Usual care (no intervention)	16	MVIC, knee extensor muscles (N)	6 MWT (m)	-	Fatigue CIS checklist
Okkersen et al. (36)	France, Germany, Netherlands, UK	E: 44.8 ± 11.7 C: 46.4 ± 11.3	255	MyD type 1	E: Cognitive behavioral and graded exercise C: Usual care	44	-	6 MWT (m)	-	Fatigue CIS checklist
NCT02421523	United States	Recruitment completed	Actual enrollment 18^***^	DMD	E1:Exercise group E2: Exercise dosing C: Usual care	12	Isokinetic peak torque, knee extensor and knee flexor muscles (Nm)		Stairs, climbing	
NCT01116570	France	Recruitment completed	Actual enrollment 15^****^	FSHD	E:Physical training on ergocycle C:Usual care	24				

*E, Experimental; C, Control; 6MWT, 6-min walk test; AET, Aerobic exercise training; AMPS, Assessment of motor and process skills; RPE, BORG rating of perceived exertion; CIS, Checklist individual strength; FSS, Fatigue severity scale; FTSTS, Five times sit to stand test; MFM, Motor function measure; MVIC, Maximum voluntary isometric contraction; TST, The timed-stands test; TUG, Timed Up and Go test; N, Newton; Nm, Moment*.

* *Mean (IQR)*.

** *Median (IQR)*.

*** *Original estimate n = 32*.

**** *Original estimate n = 3*.

### Primary Outcomes

In order to complete the overall meta-analyses, we obtained unpublished data for several trials from the corresponding authors (30, 31, 34, 35, 39).

**Figure 1:**
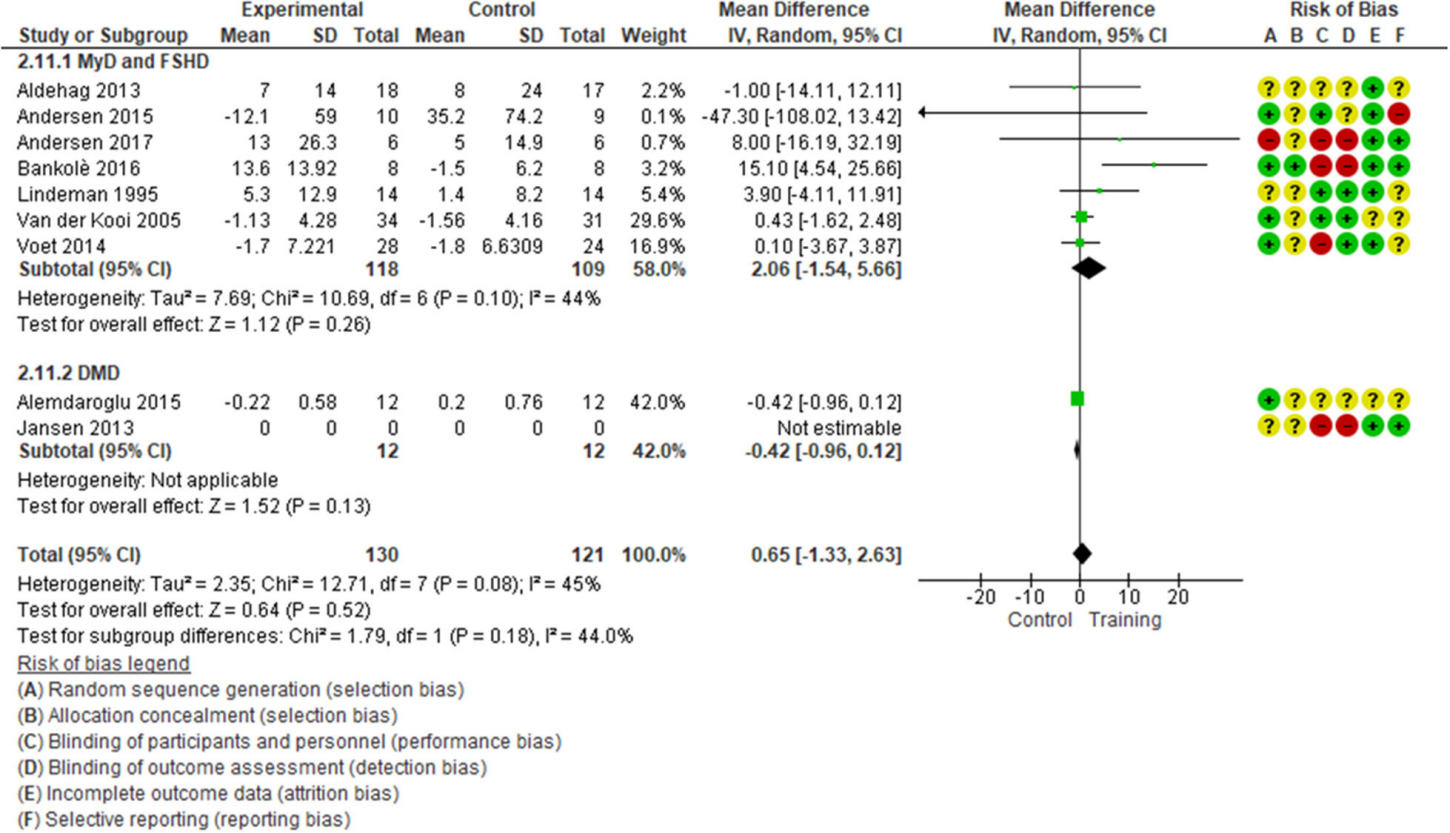
Global muscle strength, mean change in SMD for FSHD, MyD, and DMD patients. Contribution: knee extensors: Andersen et al. (34), Bankolè et al. (24), Lindeman et al. (28), Voet et al. (32); elbow flexors: Van der Kooi (40), Alemdaroglu et al. (33); wrist flexors: Aldehag et al. (37). *Alemdaroglu et al. (33) compared two training regimes (supervised training vs. unsupervised training at home).

Albeit with low quality of evidence (Table 2), the effect of muscular exercise on global muscle strength (MyD, FSHD, DMD) was not statistically different between the groups (7 studies, 239 patients, median follow-up of 16 weeks with IQR 12–24, MD 0.65; 95% CI −1.33–2.63, *I*^2^ = 45%; *p* = 0.52, Figure 1). Subgroup analysis, with very low quality of evidence (Table 2), showed no significant difference in extensor muscle strength between the groups (4 studies, 115 patients with FSHD and MyD, median follow-up of 20 weeks with IQR 15–24, MD 4.34; 95% CI −4.20 to 12.88, *I*^2^ = 69%; *p* = 0.32, eFigure S3). Sensitivity analysis, which excluded unpublished data, revealed no statistically significant differences between the groups (eFigure S4). TSA showed that the required information size of 594 patients was not achieved and that the cumulative z-curve did not cross any boundaries in favor of muscular exercise (eFigure S5). No statistically significant differences were found for knee flexors and elbow flexors (eFigures S6, S7).

**Table 2:** Quality of evidence, GRADE approach.

	**Certainty assessment**							**No of patients**		**Effect**		**Certainty**
**Outcome**	**No of studies**	**Study design**	**Risk of bias**	**Inconsistency**	**Indirectness**	**Imprecision**	**Other considerations**	**Exercise**	**Control (mean change measures)**	**Relative (95% CI)**	**Absolute (95% CI)**	
Muscle Strength	9	RCTs	Not serious	Not serious	Not serious	Serious^d^	Publication bias suspected^b^	124	115		SMD 0.03 higher (0.33 lower to 0.38 higher)	⊕⊕○○ LOW
Knee extensors muscle strength	4	RCTs	Not serious	Not serious	Serious^a^	Very serious^d^	Publication bias suspected^b^	60	55		MD 4.34 higher (−4.21 higher to 12.91 higher)	⊕○○○ VERY LOW
Endurance	5	RCTs	Not serious	Not serious	Serious^a^	Not serious	Publication bias suspected^b^	191	182	-	MD 17.36 higher (10.91 higher to 23.81 higher)	⊕⊕○○ LOW
Motor Abilities^*^	-	-	-	-	-	-	-	-	-	-	-	-
Fatigue	5	RCTs	Not serious	Serious^c^	Serious^a^	Serious^d^	No publication bias suspected	191	182		MD −0.56 lower (−1.25 lower to 0.13 higher)	⊕○○○ VERY LOW

*CI, Confidence interval; MD, Mean difference; RCTs, Randomized Controlled Trials*.

*Risk of bias was mainly weighted for selection and detection of high risk bias*.

a *Outcome only available for dystrophies with onset in adults, such as MyD and FSHD*.

b *Outcome was planned but no results are available*.

c *I^2^ > 75%*.

d *Required information size not reached and/or very large CI for the overall estimate*.

* *Not possible to meta-analyze effect sizes because the studies were too heterogeneous*.

**Figure 2:**
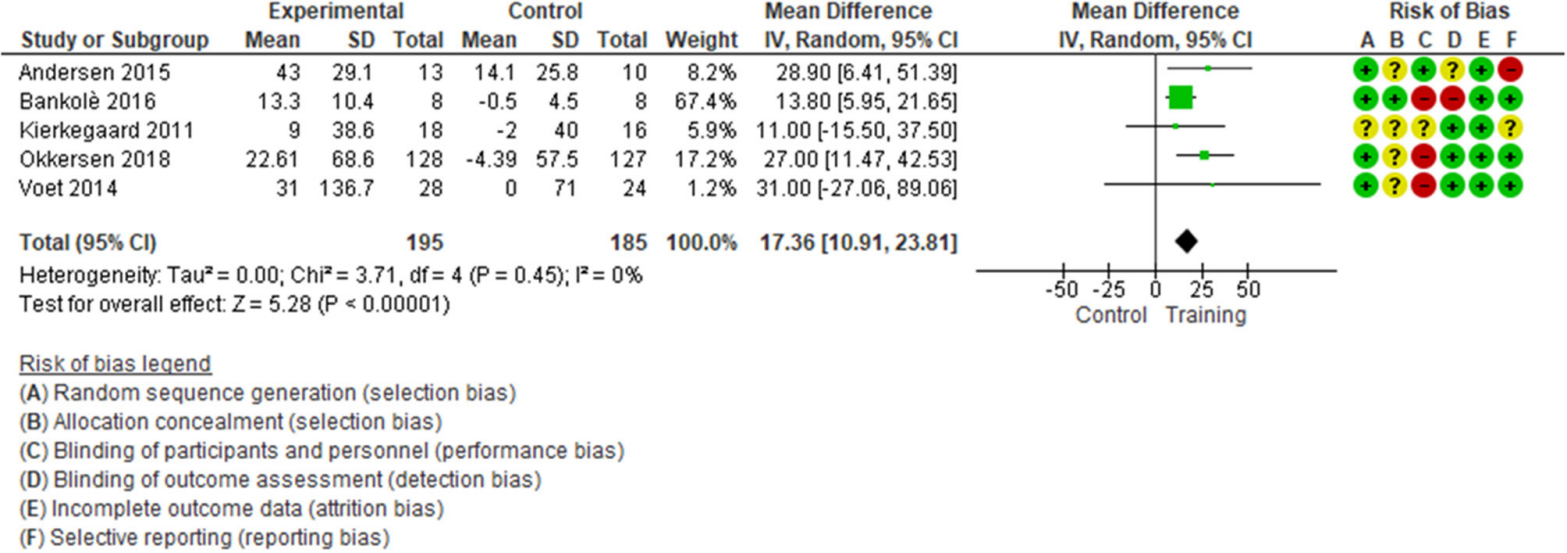
Endurance, mean change in FSHD and MyD patients.

With low quality of evidence (Table 2), we found a statistically significant difference in favor of muscular exercise for improving endurance during walking (5 studies, 380 patients with FSHD and MyD, median follow-up of 16 weeks, with IQR 14–24, MD 17.36; 95% CI 10.91–23.81, *I*^2^ = 0; *p* < 0.0001, Figure 2), TSA showed that the required information size of 110 patients was achieved; the cumulative z-curve crossed the required information size after the second study and crossed the conventional boundary at the fourth study in favor of exercise (eFigure S8). Sensitivity analysis performed only on a low risk of bias assessment for the detection of bias reinforced the statistically significant difference in favor of the exercise group (2 studies, 289 patients, MD 22.75; 95% CI 8.90–36.60, *I*^2^ = 0; *p* < 0.0001, eFigure S9). The magnitude of benefits did not reach the MID threshold set at 34.3 m.

### Secondary Outcomes

Clinical heterogeneity precluded comparison of cumulative effect size across studies on motor abilities as the tests evaluated different constructs. With very low quality of evidence, muscular exercise was not statistically significant at the end of treatment in reducing fatigue when compared to controls (five studies, 373 patients, median follow-up of 16 weeks with IQR 14–24, SMD −0.56; 95% CI −1.25–0.13, *I*^2^ = 83; *p* = 0.11, eFigure S10).

### Adverse Events

Three studies did not report information on adverse events (24, 33, 35). Six studies reported no serious adverse events (28–31, 34, 37). One study reported mild adverse effects that according to the authors did not influence the effect of the interventions (32). A multicenter trial reported 47 serious adverse events involving 34 out of 255 patients during the study (24 classified as serious in the training group and 23 in the control group), the most common of which were gastrointestinal or cardiac in nature (36).

## Discussion

In 2013 we reviewed the evidence for the possible beneficial effects of muscular exercise on patients with muscular dystrophy. At the time, we were unable to provide a clear answer due to the paucity of trials and the overall effect being equally positive or negative. Exercise might have been useful, not useful or even detrimental. The present update includes the previously reviewed studies plus later studies. We found that while muscular exercise is not associated with an improvement in strength it does improve endurance during walking compared to no treatment in patients with FSHD and MyD. The magnitude of benefit does not reach a clinically relevant threshold. However, judging the clinical meaningfulness and effect size of the mean differences (MDs) was not straightforward, since there are no internationally agreed standards (41). No conclusions can be drawn whether exercise improves motor ability since different studies used different clinical evaluations. Finally, exercise was not associated with improvements in reducing fatigue. The currently available evidence overwhelmingly suggests that exercise, while not conferring muscular protection, does not seem to be counterproductive.

The role of muscular exercise is controversial. The question whether exercise should be recommended in people with muscular dystrophy has two biologically plausible yet conflicting answers. Since a hallmark of this degenerative disease is the progressive loss of motor unit constituents, muscular exercise may be considered harmful because it can induce extensive damage, inflammation, and failure of skeletal muscle to repair itself (42). By the same token, the lack of physical activity, common in patients with muscular dystrophy, may lead to functional deconditioning, overweight, fatigue, and reduced muscle strength that could be countered.by regular physical exercise (43). Additional benefits of exercise include improvement in body composition, metabolism, cardiorespiratory performance, and mental well-being (44).

In light of current evidence and the controversy surrounding physical activity, muscle exercises should be planned neither with the aim of improving strength nor of reducing fatigue. Physiotherapists and physicians should focus their efforts on endurance training during walking. We found the similar amount of improvement reported in other degenerative disease such as the late-onset Pompe's disease (45) and mitochondrial myopathy (46).

The primary patient population is people with FSMS and MyD, probably because less impaired when performing this motor ability (13). No evidence was found for endurance training during walking in patients with DMD, however, probably because it is harder to involve this population in trials exploring this specific skill. If there are benefits, they are likely to be small. In brief, the risk of potentially detrimental effects associated with exercise is low if exercise is paced and performed gradually.

Two important issues concerning muscular exercise were not addressed in any of the studies in this review. First, exercise dose and intensity and session duration were not described accurately. These dimensions need to be carefully considered in order to fully understand how specific effects are produced, similar to when medications are prescribed. As major individual differences at different training intensities and at different dose-tolerated levels of exercise among patients with other muscle diseases have been revealed (47), new research hypotheses addressing optimal exercise prescription should be pursued in patients with muscular dystrophy, as well (48). Second, muscle exercise is rarely described as part of well-coordinated multidisciplinary program (48): current treatments usually include drugs, ventilation, and surgery, but muscle exercise is never offered within an integrated model. New research investigating multidisciplinary approaches need to include muscular exercise as a valid support to medical care.

A recent review on the topic (41) included studies perfectly overlapping ours, except for only one study (36). The quality of evidence was low in both reviews. The effects of exercise on strength remained uncertain, while there was some evidence for improved endurance during walking, which is shared by our findings.

### Limitations

This review has several limitations. First, we investigated outcome measures (endurance and strength) that capture only a snapshot of the progression of the disorder and not are applicable to all disease stages, thus limiting the number of patients included (49). Second, most of the studies had a small sample, which raises the risk of overestimating treatment effects and reducing precision. Third, some of the studies reported high drop-outs rates and low adherence to the exercise intervention, possibly reducing its treatment effects. Fourth, additional benefits derived from regular interaction with physiotherapists might have induced further distortion of treatment effects compared to the no-treatment groups. Fifth, MIDs were rarely reported, limiting the clinical interpretation of the effects of muscular exercise.

## Conclusion

Muscular exercise can be recommended to improve endurance during walking in most patients with muscular dystrophy. It is important, however, that the patient understands that the benefits might be only marginal. Indeed, realistic expectations are key to fostering cooperation between the patient and the health care staff. Muscular exercise is not recommended for strength improvement, management of motor abilities or fatigue reduction. Uncertainties in muscular exercise prescription and planning, as well as its role within multidisciplinary approaches remain. Future trials should explore which type of muscle exercise could lead to better improvements in muscle strength besides, which type of exercise lead to improvements in endurance and aerobic capacity. Well-designed trials are desirable to clarify these open issues.

## Data Availability Statement

The datasets presented in this study can be found in online repositories. The names of the repository/repositories and accession number (s) can be found below: https://osf.io/tx9d7.

## Author Contributions

SG conceived and drafted the manuscript, carried out the literature search, conducted screenings, extracted data, completed the risk of bias assessment, and performed the statistical analyses. VP provided a critical revision of the manuscript, conducted screenings, extracted data, completed the risk of bias assessment, and performed the statistical analyses. GC provided a critical revision of the manuscript, performed the statistical analyses, and assisted in writing the discussion. MM and GB interpreted the data and revised the manuscript for important intellectual content. LM conceived, interpreted the data, provided a critical revision of the manuscript, and he is the guarantor of the review. All authors contributed to the article and approved the submitted version.

## Conflict of Interest

The authors declare that the research was conducted in the absence of any commercial or financial relationships that could be construed as a potential conflict of interest.

## Footnotes

^1^Tool GGD. *GRADEpro GDT*. McMaster University (Developed by Evidence Prime, Inc.) (2015). Available online at: https://gradepro.org/cite.html (accessed: March 7, 2019).

^2^“Review Manager (RevMan) [Computer program]. Version 5.3. *Copenhagen: The Nordic Cochrane Centre, The Cochrane Collaboration* (2014). Available online at: http://community.cochrane.org/tools/review-production-tools/revman-5/about-revman-5 (accessed: March 7, 2019).

## Abbreviations

BMD: Becker's Muscular Dystrophy
CI: Confidence Intervals
DARIS: Diversity Adjusted Required Information Size
DMD: Duchenne's Muscular Dystrophy
FSHD: Facio-Scapulo-Humeral Dystrophy
LD: Limb-girdle Dystrophy
GRADE, Grading of Recommendations, Assessment: Development and Evaluations
IQR: Inter Quartile Range
MD: Mean Difference
MID: Minimal Important Difference
MyD: Myotonic Dystrophy
PRISMA: Preferred Reporting Items for Systematic Reviews and Meta-Analyses
RCT: Randomized Clinical Trials
TIDieR, Template for Intervention Description and Replication TSA: Trial Sequential Analyses
SMD: Standardized Mean Difference
6MWT: Six Minute Walking Test.
