# Supplementary material for: Effect of Muscular Exercise on Patients With Muscular Dystrophy: A Systematic Review and Meta-Analysis of the Literature
Source: Front Neurol. 2020 Nov 12;11:958. doi: 10.3389/fneur.2020.00958 (PMC7688624; doi:10.3389/fneur.2020.00958)
Supplement: Supplementary file 1 [file Data_Sheet_1.docx]

Supplementary Material

**Supplementary Figures**

**eFigure S1.** Study Flow of Literature Search.

**eFigure S2.** Risk of Bias Results.

**eFigure S3.** Mean Change in Strength of Knee Extensors in MyD and FSHD Patients.

**eFigure S4.** Sensitivity Analysis, excluding high risk of bias and unpublished data on Mean Change in Strength of Knee Extensors (Nm) in MyD and FSHD Patients.

**eFigure S5.** TSA of Strength of Knee Extensors in FSHD and MyD Patients.

**eFigure S6.** Mean Change in Strength of Knee Flexors in MyD and FSHD Patients.

**eFigure S7.** Mean Change in Strength of Elbow Flexors in MyD, FSHD and DMD Patients.

**eFigure S8**. TSA of Endurance in FSHD and MyD Patients.

**eFigure S9**. Sensitivity Analysis, excluding high risk of bias and unpublished data on Endurance in FSHD and MyD Patients.

**eFigure S10.** Mean Change in Fatigue in MyD and FSHD Patients.

**Supplementary Tables**

**eTable S1**. List of excluded studies.

**eTable S2.** Details of experimental interventions.

**Checklist 1.** PRISMA checklist.

Records retrieved in **Clinicaltrials.gov** search
(n =**35**)

Records retrieved in **PEDRO** search
(n =**15**)

Records retrieved in **WEB OF SCIENCE** search
(n =**1750**)

Records retrieved in **MEDLINE** search
(n =**1348**)

Records retrieved in **SCOPUS** search
(n =**1354**)

Records retrieved in **EMBASE** search
(n =**2656**)

Records screened
(n =**1308**)

Records after duplicates removed
(n = **5528**)

Records excluded (**n =4220**)

Full-text articles excluded, with reasons (**n =60**):

- wrong study design (n=10)

-protocol/abstract related to publication (n =9)

- ineligible outcome (n=1)

- ineligible comparator (n=2)

- ineligible population (n=1)

- ineligible intervention (n=4)

- ongoing trials not focused on exercise intervention for dystrophy (n=33)

Full-text articles assessed for eligibility
(**n =73**)

Ongoing trials without published results **(n =2)**

Studies included in quantitative synthesis
(**n=11**)

Studies included in qualitative synthesis
(**n =13**)

**eFigure S1.** Study Flow of Literature Search.

**
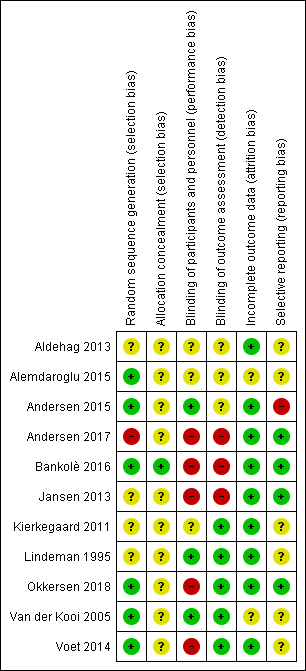
**

**eFigure S2.** Risk of Bias Results (RoB).

Legend: Red (-) = high RoB; Yellow (?) = unknown RoB; Green (+) = low RoB

**
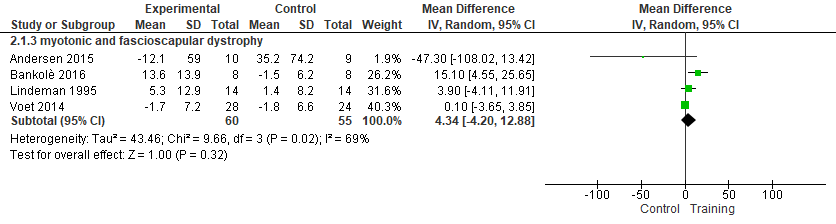
**

**eFigure S3.** Mean Change in Strength of Knee Extensors in MyD and FSHD Patients.

**
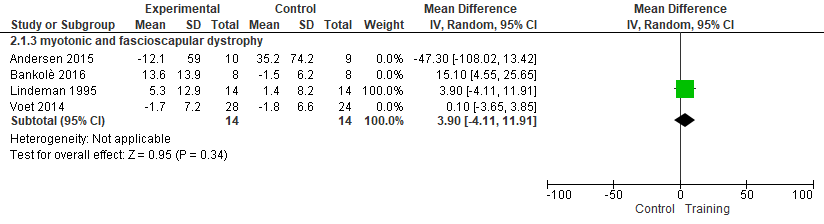
 eFigure S4.** Sensitivity Analysis, excluding high risk of bias and unpublished data on Mean Change in Strength of Knee Extensors (Nm) in MyD and FSHD Patients.

**
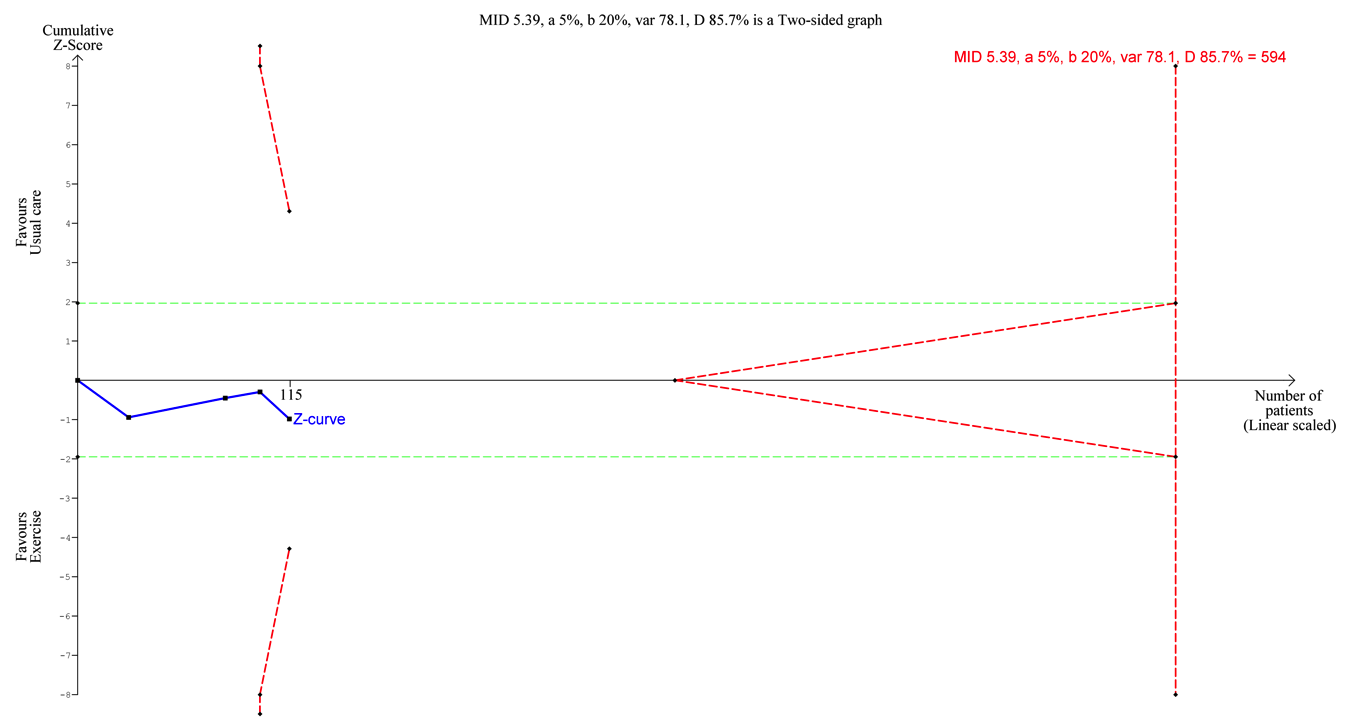
**

**eFigure S5.** TSA of Strength of Knee Extensors in FSHD and MyD Patients.


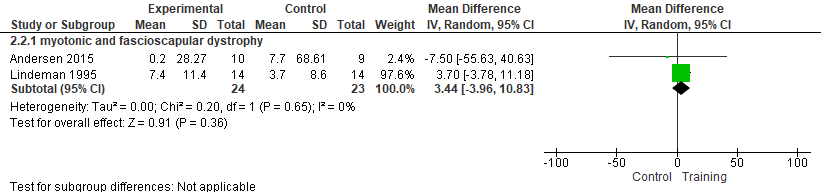


**eFigure S6.** Mean Change in Strength Knee Flexors in MyD and FSHD patients.


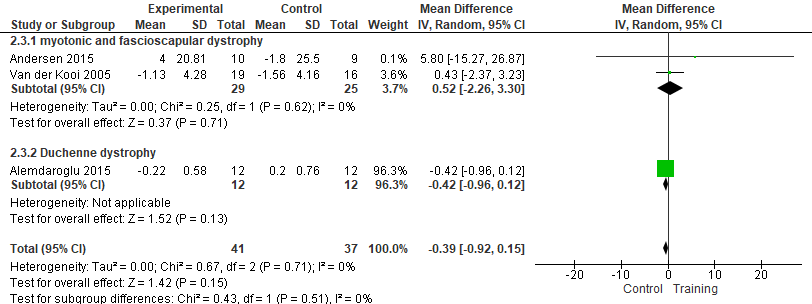
 **eFigure S7.** Mean Change in Strength of Elbow Flexors in MyD, FSHD, and DMD Patients.


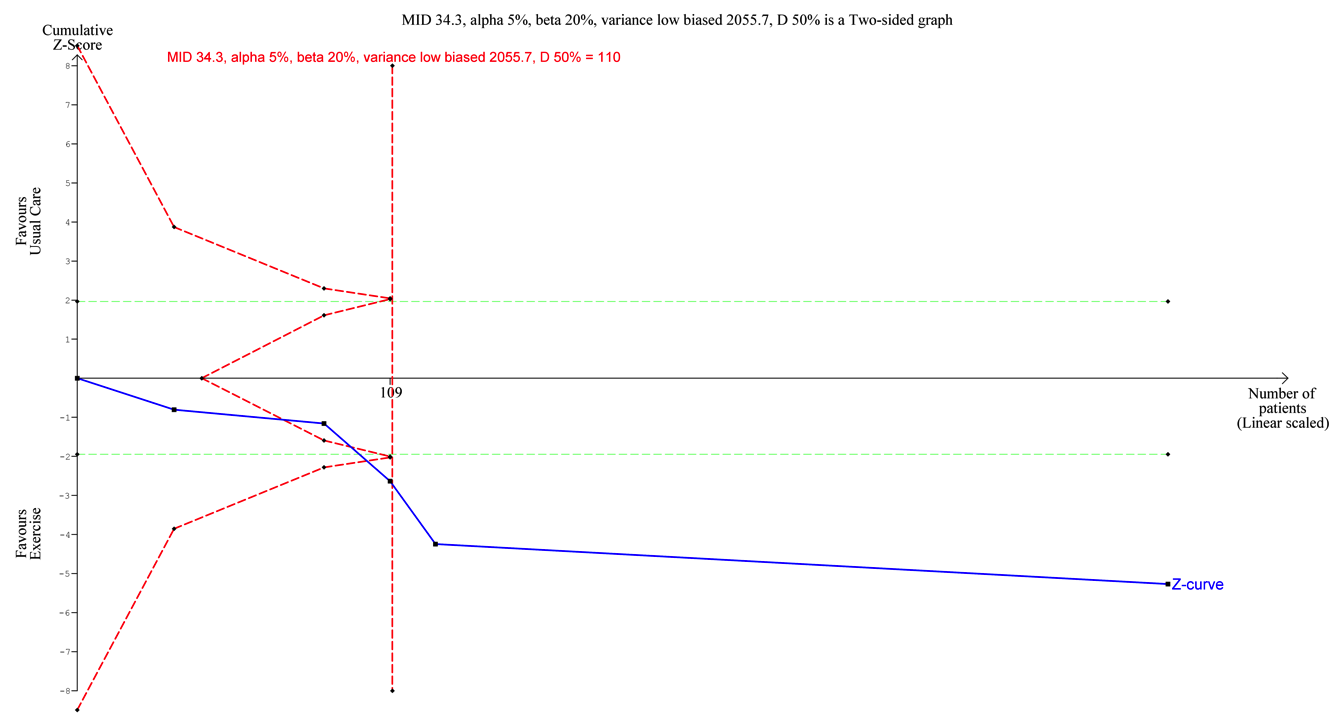


**eFigure S8**. TSA of Endurance in FSHD and MyD Patients.

**
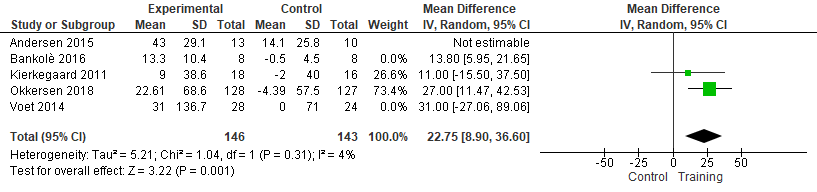
 eFigure S9**. Sensitivity Analysis, excluding high risk of bias and unpublished data on Endurance in FSHD and MyD Patients.


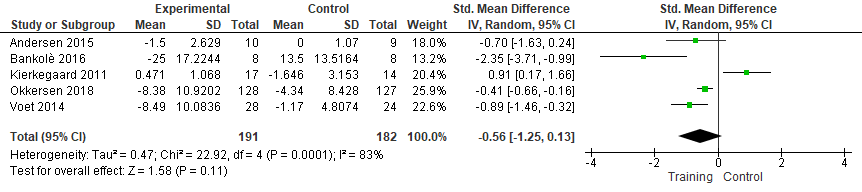


**eFigure S10.** Mean Change in Fatigue in MyD and FSHD Patients.

**eTable S1a**. List of excluded published studies (n=27).

| REFERENCES | REASON | DESCRIPTION |
| --- | --- | --- |
| Alemdaroglu, I., A. A. Karaduman, et al. (2013). "Effects of upper extremity exercise training on respiratory function and quality of life in children with Duchenne Muscular Dystrophy." Neuromuscular Disorders 23(9-10): 779. | abstract/duplicate |  |
| Alemdaroʇlu, I., A. Karaduman, et al. (2014). "Effects of upper extremity dynamic exercise on respiratory function and quality of life in Duchenne Muscular Dystrophy." Fizyoterapi Rehabilitasyon 25(2): 78-85 | abstract/duplicate |  |
| Andersen, G., K. P. Prahm, et al. (2013). "Does endurance training and protein supplementation improve fitness in patients with Facioscapulohumeral Muscle Dystrophy (FSHD)?" Neuromuscular Disorders 23(9-10): 824-825 | abstract/duplicate |  |
| Andersen, S . P., M. L. Sveen, et al. (2013). "CREATINE KINASE RESPONSE TO HIGH-INTENSITY AEROBIC EXERCISE IN ADULT-ONSET MUSCULAR DYSTROPHY." Muscle & Nerve 48(6): 897-901 | wrong study design | observational study; no intervention |
| Bankole, L. C ., G. Y. Millet, et al. (2016). "Safety and efficacy of a 6-month home-based exercise program in patients with facioscapulohumeral muscular dystrophy A randomized controlled trial." Medicine 95(31). | abstract/duplicate |  |
| Bartels, B., T. Takken, et al. (2015). "Cardiopulmonary Exercise Testing in Children and Adolescents With Dystrophinopathies: A Pilot Study." Pediatric Physical Therapy 27(3): 227-234 . | wrong intervention | test o training? |
| Berthelsen, M. P., E. Husu, et al. (2014). "Anti-gravity training improves walking capacity and postural balance in patients with muscular dystrophy." Neuromuscular Disorders 24(6): 492-498. | wrong study design | observational study |
| Brady, L. I., L. G. MacNeil, et al. (2014). "Impact of Habitual Exercise on the Strength of Individuals with Myotonic Dystrophy Type 1." American Journal of Physical Medicine & Rehabilitation 93(9): 739-746. | wrong study design | retrospective study |
| Buch, A., G. Andersen, et al. (2015). "High intensity training in patients with facioscapulohumeral muscular dystrophy." Neuromuscular Disorders 25((Buch A.) Rigshospitalet, University Hospital of Copenhagen, Copenhagen Neuromuscular Center, Rigshopsitalet, Denmark): S215. | wrong control | healthy |
| Cudia, P., L. Weis, et al. (2016). "Effects of Functional Electrical Stimulation Lower Extremity Training in Myotonic Dystrophy Type I: A Pilot Controlled Study." American Journal of Physical Medicine & Rehabilitation 95(11): 809-817. | wrong intervention | Electrical Stimulation in controlled but not randomized trial: RCT that makes a comparison between 2 different training regimes. No comparison of training versus non-training participants |
| Hammarén, E., C. Lindberg, et al. (2015). "Effects of a balance exercise programme in myotonic dystrophy type 1: A pilot study." European Journal of Physiotherapy 17(3): 123-131 . | wrong control | before and after a 10 week intervention phase and after a 12 week follow-up phase. Each subject was his or her own control. |
| Hedermann, G., C. R. Vissing, et al. (2016). "Aerobic Training in Patients with Congenital Myopathy." Plos One 11(1). | wrong population |  |
| Jensen, B. R., M. P. Berthelsen, et al. (2016). "BODY WEIGHT-SUPPORTED TRAINING IN BECKER AND LIMB GIRDLE 2I MUSCULAR DYSTROPHY." Muscle & Nerve 54(2): 239-243. | wrong study design | observational study: 1 cohort without control |
| Kilinc, M. and S. A. Yildirim (2015). "The effects of electrical stimulation and exercise therapy in patients with limb girdle muscular dystrophy A controlled clinical trial." Neurosciences 20(3): 259-266. | wrong intervention | controlled not randomized that makes a comparison between 2 different training regimes. No comparison of training versus non-training participants |
| Pandya, S., J. Andrews, et al. (2016) | wrong intervention |  |
| Sveen, M. L., S. P. Andersen, et al. (2013). "Resistance training in patients with limb-girdle and becker muscular dystrophies." Muscle & Nerve 47(2): 163-169. | wrong study design | observational study |
| Van Engelen, B. G., N. Voet, et al. (2014). "Aerobic exercise and cognitive behavior therapy reduce fatigue and slow progression of muscle MR fatty infiltration in FSHD." Neuromuscular Disorders 24(9-10): 798-799. | abstract/duplicate |  |
| Voet, N., G. Bleijenberg, et al. (2014). "Both aerobic exercise training and cognitive behavior therapy reduce chronic fatigue in patients with facioscapulohumeral muscular dystrophy: A randomized controlled trial." Annals of Physical and Rehabilitation Medicine 57((Voet N.; Bleijenberg G.; De Groot I.; Padberg G.; Van Engelen B.; Geurts A.) Radboud UMC, Nijmegen, Netherlands): e96. | abstract/duplicate |  |
| Voet, N. B. M., G. Bleijenberg, et al. (2015). "Both aerobic exercise and cognitive-behavioral therapy reduce fatigue in FSHD: An RCT." Nederlands Tijdschrift voor Geneeskunde 159(12) | abstract/duplicate |  |
| 1701.1702. Wang, L., S. L. Katz, et al. (2014). "Stacking exercises aid the decline in FVC and sick time (STEADFAST) in Duchenne muscular dystrophy: A pilot study." Canadian Journal of Neurological Sciences 41((Wang L.; Mah J.K.) Calgary, Canada): S15 | wrong outcome of interest | respiratory |
| Heje, K., G. Andersen, et al. (2015). "High intensity training in patients with spinal and bulbar muscular atrophy." Neuromuscular Disorders 25((Heje K.; Andersen G.; Vissing J.) Rigshospitalet, University Hospital, Copenhagen, Denmark): S225 | abstract/duplicate |  |
| Voet, N. B., E. L. van der Kooi, et al. (2013). "Strength training and aerobic exercise training for muscle disease." Cochrane Database Syst Rev(7): CD003907. | wrong study design | review |
| Voet, N. (2014). "Update on the scientific evidence for exercise in neuromuscular diseases." Annals of Physical and Rehabilitation Medicine 57((Voet N.) Radboud University Medical Centre, Nijmegen, Netherlands): e96. | abstract/duplicate | review |
| Anziska,  Y. and S. Inan (2014). "Exercise in Neuromuscular Disease." Seminars in Neurology 34(5): 542-556. | wrong study design | review |
| Anziska, Y. and A. Sternberg (2013). "Exercise in neuromuscular disease." Muscle & Nerve 48(1): 3-20. | wrong study design | review |
| Corrado, B. and G. Ciardi (2015). "Facioscapulohumeral distrophy and physiotherapy: a literary review." Journal of Physical Therapy Science 27(7): 2381-2385. | wrong study design | review |
| Dahlqvist, J. R. and J. Vissing (2016). "Exercise Therapy in Spinobulbar Muscular Atrophy and Other Neuromuscular Disorders." Journal of Molecular Neuroscience 58(3): 388-393. | wrong study design | review |

**eTable S1b**. List of excluded ongoing trials (n=33).

| Rank | NCT Number | Status | Study Results | Conditions | EXCLUDED |
| --- | --- | --- | --- | --- | --- |
| 1 | NCT04173234 | Recruiting | No Results Available | Duchenne Muscular Dystrophy | wrong intervention |
| 2 | NCT02158156 | Unknown status | No Results Available | Oculopharyngeal Muscular Dystrophy | wrong population |
| 3 | NCT02159963 | Completed | No Results Available | FSHD - Facioscapulohumeral Muscular Dystrophy | already included |
| 4 | NCT04187482 | Active, not recruiting | No Results Available | Myotonic Dystrophy 1\|Muscular Dystrophies | no control |
| 5 | NCT02641275 | Withdrawn | No Results Available | Muscular Dystrophies | wrong population |
| 6 | NCT01618331 | Completed | No Results Available | Facioscapulohumeral Muscle Dystrophy | already included |
| 7 | NCT01895283 | Completed | No Results Available | Bethlem Myopathy | wrong population |
| 8 | NCT01999075 | Completed | No Results Available | Duchenne Muscular Dystrophy | wrong intervention |
| 9 | NCT04392518 | Recruiting | No Results Available | Telemedicine\|Muscular Dystrophies\|Proximal Myopathy | wrong intervention |
| 10 | NCT02614820 | Unknown status | No Results Available | Duchenne Muscular Dystrophy (DMD) | wrong intervention |
| 11 | NCT04009408 | Not yet recruiting | No Results Available | Oculopharyngeal Muscular Dystrophy\|Muscular Dystrophies\|Myopathy | wrong population |
| 12 | NCT03879304 | Enrolling by invitation | No Results Available | Muscular Dystrophy, Duchenne and Becker Types | wrong intervention |
| 13 | NCT02653833 | Terminated | No Results Available | Muscular Dystrophy | wrong intervention |
| 14 | NCT02147639 | Completed | No Results Available | Becker Muscular Dystrophy | wrong intervention |
| 15 | NCT03689660 | Not yet recruiting | No Results Available | Neuromuscular Disease\|Duchenne Muscular Dystrophy\|Spinal Muscular Atrophy\|Virtual Reality\|Biofeedback | wrong intervention |
| 16 | NCT01856868 | Completed | No Results Available | Becker Muscular Dystrophy | wrong intervention |
| 17 | NCT03236662 | Completed | No Results Available | Becker Muscular Dystrophy | wrong intervention |
| 18 | NCT00821548 | Completed | No Results Available | Facioscapulohumeral Muscular Dystrophy | wrong intervention |
| 19 | NCT00866112 | Completed | No Results Available | Spinal Cord Injury\|Multiple Sclerosis\|Muscular Dystrophy\|Cerebral Palsy\|Spina Bifida\|Amputation\|Arthritis\|Stroke | wrong population |
| 20 | NCT04461561 | Not yet recruiting | No Results Available | Cancer\|Cardiac Anomaly\|Cystic Fibrosis\|Muscular Dystrophy\|HIV/AIDS\|Batten's Disease\|Cerebral Palsy | wrong population |
| 21 | NCT02168114 | Completed | No Results Available | Duchenne Muscular Dystrophy | wrong intervention |
| 22 | NCT03127241 | Completed | No Results Available | Muscular Dystrophies\|Muscular Dystrophy, Duchenne\|Muscular Dystrophy, Becker\|Muscular Dystrophy, Limb-Girdle Type 2 | wrong intervention |
| 23 | NCT02341053 | Unknown status | No Results Available | Spinal Muscular Dystrophy\|Neuromuscular Disability | wrong population |
| 24 | NCT04001920 | Completed | No Results Available | Myotonic Dystrophy 1 | no control |
| 25 | NCT04052958 | Enrolling by invitation | No Results Available | Myotonic Dystrophy 1 | wrong intervention |
| 26 | NCT01359670 | Completed | No Results Available | Duchenne Muscular Dystrophy | wrong intervention |
| 27 | NCT04018820 | Completed | No Results Available | Myotonic Dystrophy 1 | no control |
| 28 | NCT02428673 | Active, not recruiting | No Results Available | Osteopenia\|Spinal Muscular Atrophy\|Cerebral Palsy\|Muscular Dystrophy\|Spina Bifida\|Rett Syndrome | wrong population |
| 29 | NCT02635269 | Active, not recruiting | No Results Available | Metabolism, Inborn Errors\|Lipid Metabolism, Inborn Errors\| | wrong population |
| 30 | NCT03642860 | Recruiting | No Results Available | Tarui Disease\|Debrancher Deficiency\|GYG1 DEFICIENCY | wrong population |
| 31 | NCT02118779 | Completed | No Results Available | Myotonic Dystrophy Type 1 | wrong intervention |
| 32 | NCT02861911 | Terminated | No Results Available | Primary Disease Facioscapulohumeral Dystrophy (FSHD) | wrong intervention |
| 33 | NCT03653390 | Recruiting | No Results Available | Physical Disability | wrong population |

**eTable S2.** Characteristics of experimental interventions.

| **Study, year** | **Type of training**  **and exercise** | **Intensity** | **Frequency** | **Setting** | **Duration** | **Muscle groups trained** | **Supervision** |
| --- | --- | --- | --- | --- | --- | --- | --- |
| Lindeman  1995 | Dynamic strength training with weights | Individualized progressive overload, 3 sets with 25 repetitions at 60% 1 RM, via 15 repetitions at 70%, to 10 repetitions at 80% | 3 times/week | At home (the Netherlands) | Session: within 30 min. Duration: 24 weeks | Knee extensors and flexors, hip extensors and abductors | Supervised home training program |
| Van der Kooi  2004 and 2007 | Dynamic and isometric strength training with weights | Individualized progressive overload, 2 sets dynamic from 10 repetitions at 10 RM, via 8 repetitions at 8 RM, to 5 repetitions at 5 RM, and 30 s isometric with same weight | 3 times/week | At home (the Netherlands) | Session: within 30 min. Duration: 52 weeks | Elbow flexors, ankle dorsiflexors | Supervised at home by physical therapist |
| Kierkegaard  2011 | Strength training, aerobic exercise, balance exercises accompanied by music | Strength exercises for arm, leg, back, and abdominal muscles 16-20 repetitions for 6-7 min, balance exercises for 3-4 min, aerobic activities for 11-12 min at 60%-80% maximum heart rate. 30-min brisk walk once a week | 2 times/week and a brisk walk once a week | Department of Physical Therapy, Karolinska University Hospital (Sweden) | Session: 60 min and a 30-min walk.  Duration: 14 weeks | Arms, back, and abdomen | All sessions supervised by specialized physiotherapist |
| Aldeahag  2013 | Hand training program: dynamic strength-endurance exercises, i.e., mass wrist- and finger movements,  and isolated finger movements, and stretching exercises for wrist and finger muscles vs. no  training | Resistance of putty (supersoft to medium) according to participant’s baseline hand-grip force | 3 times/week. Number of sets for each movement progressively increased during 12-week training, one set every 4th week. Each mass movement consisted of 1 set of 10 repetitions  on weeks 1-4, 2 sets of repetitions on weeks 5-8, and 3 sets of 10 repetitions on  weeks 9-12. | Every week, 1 session performed at Department of Occupational Therapy, Karolinska University  Hospital, (Sweden). 2 sessions performed at home. | Session: approximately 1 h.  Duration: 12 weeks | Wrist and finger muscles | 1 session/week in group-training, supervised by occupational therapist. |
| Alemdaroglu  2015 | Different types of  upper extremity exercise training (exercise with  arm ergometer and ROM exercise) | Sessions began with 5 min in passive mode (warm-up), followed by 30 min in active mode, and ended with another 5 min in passive mode to cool down. | 3 times/week | Turkey | Session: 40 min. on average Duration: 8 weeks | Upper extremities | Study group exercised with  arm ergometer supervised by physiotherapist,  control group received strengthening  range-of-motion exercise supervised by r families |
| Andersen 2015 | Cycling exercises on an ergometer | 70% VO_2_ max | 3 times/week | Copenhagen Neuromuscular Center (Denmark) | Session: 15 min in first week, 20 min in second week, 30 min thereafter. Duration: 12 weeks |  | Participants kept a diary and were supervised by phone. Number of phone contacts varied,  depending on individual need for supervision. |
| Andersen 2017 | Cycling exercises on an ergometer | Each min of high-intensity training (HIT) performed at 3 different work intensities: 30 s easy pedaling, 20 s hard work, and 10 s maximal intensity. | 3 times/week | Copenhagen Neuromuscular Center (Denmark) and at home | Session: 21 min including 8-min standardized warm-up and 2 sets of 5-min HIT separated by 3-min break at very low intensity. Duration: 8 weeks |  | 1 weekly session performed in clinic. Participants received live training instructions and  recorded training guide for home use. |
| Bankole, 2016 | Assisted bicycle home training program | 2 combined sessions consisted of aerobic exercise at constant moderate intensity (60% of mean arterial pressure [MAP]) followed by steps of near-maximal revolutions. Session 3 consisted of interval training at 40% to 80% of MAP. Exercise intensity individualized based on either observed  heart rate reduction with training or new MAP from incremental cycling sessions | 3 times/week | At home (France) | Session: 35 min. Duration: 24 weeks | Upper and lower leg muscles | First 5-10 training sessions supervised by experienced exercise physiologist. Every week,  exercise physiologist provided telephone support for 2 sessions and attended 3rd session to supervise it and adjust individualized exercise intensity |
| Jensen 2013 | Assisted bicycle home training program | 15 min cycling with arms and legs using a mobility trainer with electrical motor assistance | 5 days/week | At home or at school, depending on participant preference (the Netherlands) | Session: 15 min. Duration: 24 weeks | Arm and leg muscles | Parents and/or teachers instructed to assist boys. Training intensity and posture monitored  and if necessary adjusted by primary investigator |
| Voet 2014 | Cycling exercises on an ergometer | Resistance increased until increase of 50%-65% in heart rate reserve achieved. | 3 times/week, twice a week at home, once a week at rehabilitation center | Once a week at rehabilitation department of one of the 6 participating centers (St Maartenskliniek  Nijmegen, de Hoogstraat Utrecht, de Vogellanden Zwolle, het Roessingh Enschede, St Franciscus  ziekenhuis Roosendaal, Reade Amsterdam, the Netherlands) | Session: 30 min, with additional warm-up and coo-down periods of 5 and 3 min, respectively.  Duration: 16 weeks |  | Supervised by physiotherapist |
| Okkersen  2018 | Graded  exercise module constituted a structured exercise  program for increasing physical fitness. Program individually tailored but targeted  incorporating moderate-intensity exercises (e.g., walking,  cycling, jogging or dancing) | Moderate-intensity exercises (e.g., walking, cycling, jogging, or dancing) | 3 times/week. | located in Paris (France), Munich (Germany), Nijmegen  (Netherlands), and Newcastle (UK). | Session: 30 min.  Patients received 10–14 sessions  cognitive behavioral therapy (no specific duration  specified)  Duration: 10 months |  | Minimum of five face-to-face sessions, but other  communication formats, such as telephone or video calls  acceptable. |

**Checklist 1.** PRISMA checklist.

| **Section/topic** | **#** | **Checklist item** | **Reported on page #** |
| --- | --- | --- | --- |
| **TITLE** | | |  |
| Title | 1 | Identify the report as a systematic review, meta-analysis, or both. | 1 |
| **ABSTRACT** | | |  |
| Structured summary | 2 | Provide a structured summary including, as applicable: background; objectives; data sources; study eligibility criteria, participants, and interventions; study appraisal and synthesis methods; results; limitations; conclusions and implications of key findings; systematic review registration number. | 3-4 |
| **INTRODUCTION** | | |  |
| Rationale | 3 | Describe the rationale for the review in the context of what is already known. | 5 |
| Objectives | 4 | Provide an explicit statement of questions being addressed with reference to participants, interventions, comparisons, outcomes, and study design (PICOS). | 5 |
| **METHODS** | | |  |
| Protocol and registration | 5 | Indicate if a review protocol exists, if and where it can be accessed (e.g., Web address), and, if available, provide registration information including registration number. | 6 |
| Eligibility criteria | 6 | Specify study characteristics (e.g., PICOS, length of follow-up) and report characteristics (e.g., years considered, language, publication status) used as criteria for eligibility, giving rationale. | 6 |
| Information sources | 7 | Describe all information sources (e.g., databases with dates of coverage, contact with study authors to identify additional studies) in the search and date last searched. | 6 |
| Search | 8 | Present full electronic search strategy for at least one database, including any limits used, such that it could be repeated. | 7 |
| Study selection | 9 | State the process for selecting studies (i.e., screening, eligibility, included in systematic review, and, if applicable, included in the meta-analysis). | 6-7 |
| Data collection process | 10 | Describe method of data extraction from reports (e.g., piloted forms, independently, in duplicate) and any processes for obtaining and confirming data from investigators. | 8 |
| Data items | 11 | List and define all variables for which data were sought (e.g., PICOS, funding sources) and any assumptions and simplifications made. | 6-7-8 |
| Risk of bias in individual studies | 12 | Describe methods used for assessing risk of bias of individual studies (including specification of whether this was done at the study or outcome level), and how this information is to be used in any data synthesis. | 9 |
| Summary measures | 13 | State the principal summary measures (e.g., risk ratio, difference in means). | 10 |
| Synthesis of results | 14 | Describe the methods of handling data and combining results of studies, if done, including measures of consistency (e.g., I^2^) for each meta-analysis. | 10 |
| **RESULTS** | | |  |
| Study selection | 17 | Give numbers of studies screened, assessed for eligibility, and included in the review, with reasons for exclusions at each stage, ideally with a flow diagram. | 11 |
| Study characteristics | 18 | For each study, present characteristics for which data were extracted (e.g., study size, PICOS, follow-up period) and provide the citations. | 11 |
| Risk of bias within studies | 19 | Present data on risk of bias of each study and, if available, any outcome level assessment (see item 12). | 11 and suppl |
| Results of individual studies | 20 | For all outcomes considered (benefits or harms), present, for each study: (a) simple summary data for each intervention group (b) effect estimates and confidence intervals, ideally with a forest plot. | 12-13 |
| Synthesis of results | 21 | Present results of each meta-analysis done, including confidence intervals and measures of consistency. | 12-13 |
| Risk of bias across studies | 22 | Present results of any assessment of risk of bias across studies (see Item 15). | na |
| Additional analysis | 23 | Give results of additional analyses, if done (e.g., sensitivity or subgroup analyses, meta-regression [see Item 16]). | suppl |
| **DISCUSSION** | | |  |
| Summary of evidence | 24 | Summarize the main findings including the strength of evidence for each main outcome; consider their relevance to key groups (e.g., healthcare providers, users, and policy makers). | Table 2 |
| Limitations | 25 | Discuss limitations at study and outcome level (e.g., risk of bias), and at review-level (e.g., incomplete retrieval of identified research, reporting bias). | 16 |
| Conclusions | 26 | Provide a general interpretation of the results in the context of other evidence, and implications for future research. | 17 |
| **FUNDING** | | |  |
| Funding | 27 | Describe sources of funding for the systematic review and other support (e.g., supply of data); role of funders for the systematic review. | 19 |
